# Poly[[tris­(μ_2_-acetato-κ^2^ *O*:*O*′)(4-chloro­benzene-1,2-di­amine-κ*N*)(μ_3_-hydroxido)dizinc] ethanol monosolvate]

**DOI:** 10.1107/S1600536814012641

**Published:** 2014-06-07

**Authors:** David K. Geiger, Dylan E. Parsons

**Affiliations:** a Department of Chemistry, State University of New York-College at Geneseo, 1 College Circle, Geneseo, NY 14454, USA

## Abstract

The title compound, {[Zn_2_(CH_3_CO_2_)_3_(OH)(C_6_H_7_ClN_2_)]·C_2_H_5_OH}_*n*_, has alternating octa­hedrally and tetra­hedrally coordinated Zn^2+^ ions. The octa­hedral coordination sphere is composed of one N atom of the monodentate di­amino­chloro­benzene ligand, three acetate O atoms and two bridging hydroxide ligands. The tetra­hedral coordination sphere consists of three acetate O atoms and the hydroxide ligand. The zinc ions are bridged by acetate and hydroxide ligands. The result is a laddered-chain structure parallel to [100] with ethanol solvent mol­ecules occupying the space between the chains. The di­amine ligand chlorine substitutent is disordered over two equally populated positions as a result of a crystallographically imposed inversion center between adjacent ligands. The ethanol solvent mol­ecule exhibits disorder with the two components having refined occupancies of 0.696 (11) and 0.304 (11). O—H⋯O hydrogen bonds form between the hydroxide ligand and the ethanol solvent mol­ecule. N—H⋯O and N—H⋯N hydrogen bonding between the uncoordinated amine group and the acetate ligands and the coordinated amine group are also observed.

## Related literature   

A recent review of crystalline metal-organic frameworks has been published by Dey *et al.* (2014 ▶). For a review of such compounds in chemical sensors, see: Kreno *et al.* (2012 ▶) and for a review of the synthesis of such compounds, see: Farha & Hupp (2010 ▶). For some other examples of zinc compounds with chain structures and bridging acetate ligands, see: Tan *et al.* (2011 ▶); Luo *et al.* (2011 ▶); Liu (2010 ▶); Hou *et al.* (2007*a* ▶,*b* ▶). For examples of zinc complexes with monodentate 1,2-diaminobenzene ligands, see: Geiger (2012 ▶); Ovalle-Marroquín *et al.* (2002 ▶). 
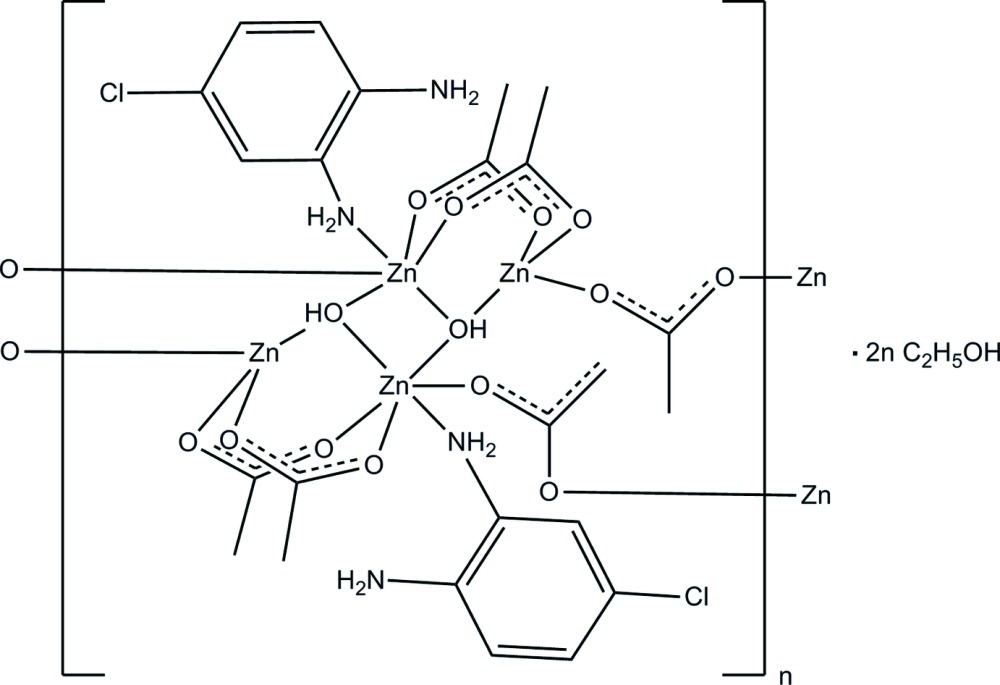


## Experimental   

### 

#### Crystal data  

[Zn_2_(C_2_H_3_O_2_)_3_(OH)(C_6_H_7_ClN_2_)]·C_2_H_6_O
*M* *_r_* = 513.53
Triclinic, 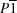

*a* = 8.0769 (12) Å
*b* = 10.8723 (19) Å
*c* = 12.909 (3) Å
α = 101.511 (6)°
β = 96.399 (6)°
γ = 109.817 (5)°
*V* = 1025.1 (3) Å^3^
*Z* = 2
Mo *K*α radiation
μ = 2.51 mm^−1^
*T* = 200 K
0.60 × 0.40 × 0.02 mm

#### Data collection  

Bruker SMART X2S benchtop diffractometer
Absorption correction: multi-scan (*SADABS*; Bruker, 2013 ▶) *T*_min_ = 0.52, *T*_max_ = 0.95
6443 measured reflections
3329 independent reflections
2403 reflections with *I* > 2σ(*I*)
*R*_int_ = 0.061

#### Refinement  

*R*[*F* ^2^ > 2σ(*F* ^2^)] = 0.059
*wR*(*F* ^2^) = 0.181
*S* = 1.05
3329 reflections
280 parameters
92 restraints
H atoms treated by a mixture of independent and constrained refinement
Δρ_max_ = 0.98 e Å^−3^
Δρ_min_ = −1.05 e Å^−3^

### 

Data collection: *APEX2* (Bruker, 2013 ▶); cell refinement: *SAINT* (Bruker, 2013 ▶); data reduction: *SAINT*; program(s) used to solve structure: *SHELXS97* (Sheldrick, 2008 ▶); program(s) used to refine structure: *SHELXL2013* (Sheldrick, 2008 ▶); molecular graphics: *PLATON* (Spek, 2009 ▶) and *Mercury* (Macrae *et al.*, 2008 ▶); software used to prepare material for publication: *publCIF* (Westrip, 2010 ▶).

## Supplementary Material

Crystal structure: contains datablock(s) global, I. DOI: 10.1107/S1600536814012641/gg2136sup1.cif

Structure factors: contains datablock(s) I. DOI: 10.1107/S1600536814012641/gg2136Isup2.hkl

Supporting information file. DOI: 10.1107/S1600536814012641/gg2136Isup4.mol

CCDC reference: 1006074

Additional supporting information:  crystallographic information; 3D view; checkCIF report

## Figures and Tables

**Table 1:** Hydrogen-bond geometry (Å, °)

*D*—H⋯*A*	*D*—H	H⋯*A*	*D*⋯*A*	*D*—H⋯*A*
O1—H1⋯O*E*1^i^	0.84 (2)	1.99 (3)	2.807 (11)	166 (8)
O1—H1⋯O*E*2^i^	0.84 (2)	2.03 (4)	2.83 (2)	160 (8)
N2—H2*A*⋯O81^ii^	0.90 (2)	2.38 (7)	2.914 (9)	118 (6)
N2—H2*B*⋯N1^iii^	0.91 (6)	2.32 (5)	3.128 (10)	148 (7)
N1—H1*A*⋯O71^iv^	0.91 (7)	2.19 (3)	3.082 (9)	169 (6)
O*E*1—H1*E*1⋯O82	0.84	1.97	2.780 (11)	163
O*E*2—H1*E*2⋯O92	0.84	2.32	3.02 (3)	140

Symmetry codes: (i) 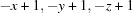; (ii) 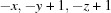; (iii) 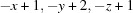; (iv) 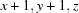.

**Fig. 1.**
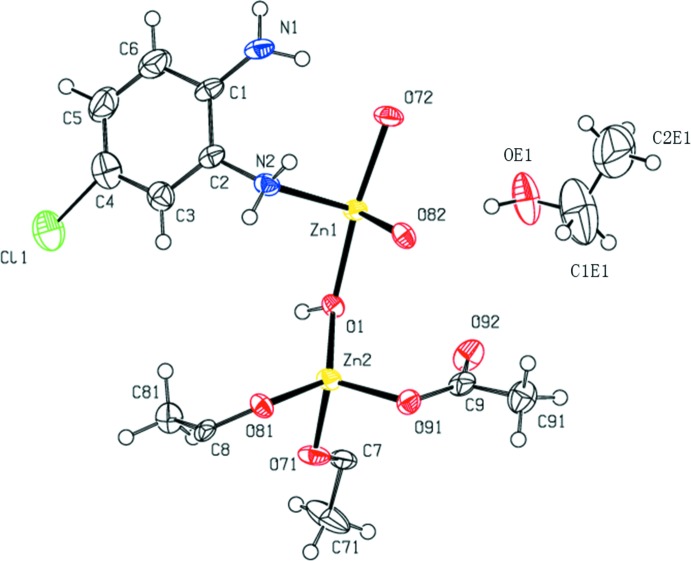
Asymmetric unit of the title compound with the atom-labeling scheme. Only the major contributor to the disordered ethanol and one of the half-occupied Cl atoms are represented. Anisotropic displacement parameters are drawn at the 50% probability level.

**Fig. 2.**
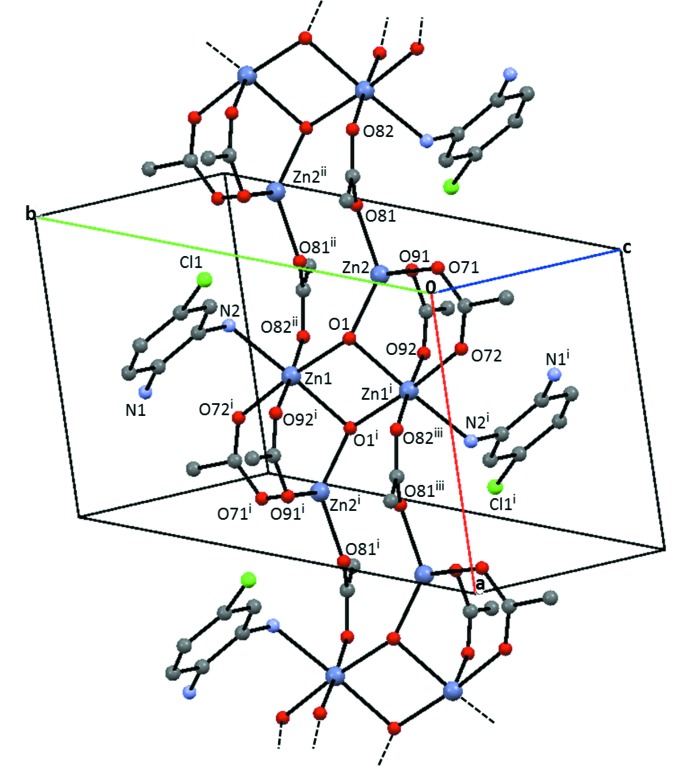
A view of the polymeric chain parallel to [1 0 0]. The ethanol solvate and hydrogen atoms are not shown for clarity. Only one of the partially occupied Cl sites is represented. Symmetry codes: (i) -x+1, -y+1, -z+1; (ii) -x, -y+1, -z + 1; (iii) x + 1, y, z.

**Fig. 3.**
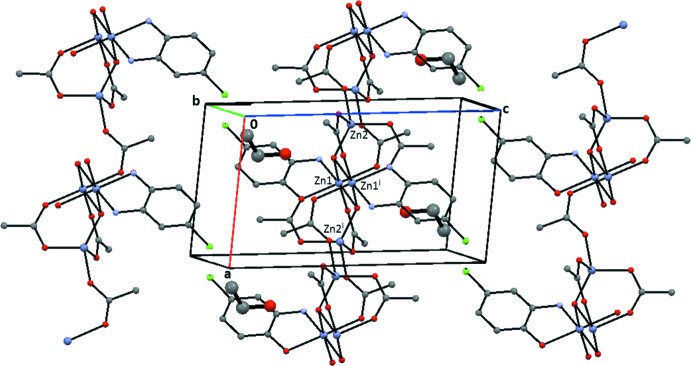
Packing diagram emphasizing the location of the ethanol solvent molecules with respect to the polymer chain. Only one of the partially occupied Cl sites is represented and only the major contributor to the disorder model is represented. Hydrogen atoms are omitted for clarity. Symmetry code: (i) -x+1, -y+1, -z+1.

## supplementary crystallographic information

### S1. Comment

Because of their large internal surface areas and uniformly structured cavities which small molecules may occupy, metal-organic frameworks (MOFs) are of inherent interest in areas such as gas storage, catalysis, chemical sensors and molecular separation (Dey *et al.*, 2014; Kreno *et al.*, 2012; Farha & Hupp, 2010). The title compound is an example of a one-dimensional MOF in which space between polymeric chains provides room for incorporation of small, non-covalently bonded molecules. To our knowledge, this compound is the first example of a mixed acetato and monodentetate diamine coordinated zinc compound with a one-dimensional chain structure.

The title compound exhibits a laddered-chain structure. The atom-labeling scheme for the basic repeating unit is shown in figure 1. Only the major contributor to the disordered ethanol solvate molecule is shown and only one of the two half-occupied sites of the chlorine substitutent is represented. (The other half-occupied chlorine is bound to C4.) The disorder of the chlorine results from a combination of a crystallographically-imposed inversion center between adjacent chlorodiaminobenzene ligands and the unsymmetrical substitution of the diamine ligand (i.e., Zn coordination of one of the nitrogen atoms puts the chlorine atom in one of the occupied sites and coordination of the other nitrogen atom puts the chlorine atom in the other partially occupied site).

Figure 2 shows a view of the polymeric chain in which the distorted octahedral and tetrahedral coordination spheres of the zinc ions are visible. Two asymmetric units make up the repeating motif, a Zn_4_(µ-OH)_2_(µ-acetato-κ^2^-O:O')_4_ core in which each of two distorted-octahedrally-coordinated Zn ions are bridged to a distorted-tetrahedrally-coordinated Zn ion by two acetate ligands and one hydroxide ligand. The octahedral coordination sphere is completed by a monodentate 1,2-diamino-4-chlorobenzene ligand and a third bridging acetato-κ^2^-O:O' ligand. The tetrahedral coordination sphere is completed by a bridging acetato-κ^2^-O:O' ligand.

Coordination to the bridging hydroxide ligands is decidedly unsymmetrical. The Zn1–O1 bond distances are 2.079 (5) Å and 2.147 (5) Å with the shorter bond distance corresponding to the hydroxide ligand *trans* to the diaminobenzene ligand (see Figure 2). The Zn2–O1 bond distance is 1.937 (5) Å. The Zn1–O1–Zn1^i^ angle is 97.7 (2)^o^ and the Zn2–O1–Zn1 and Zn2–O1–Zn1^i^ angles are 127.8 (2)^o^ and 101.7 (2)^o^. The tetrahedral coordination sphere is highly distorted. The O–Zn—O angles range from 94.7 (2)^o^ to 130.8 (2)^o^, respectively.

Space between the chains is occupied by approximately two-fold rotationally-disordered ethanol molecules (see figure 3). Calculations using PLATON (Spek, 2009) show that the ethanol solvate molecule occupies a void with a volume of 224.1 Å^3^. Single crystals of the compound were subjected to vacuum at room temperature for extended periods followed by data collection. Subsequent structure solution revealed that the solvate remained trapped in the void. Attempts to heat crystals to 110^o^C under vacuum resulted in decomposition. Hydrogen bonds between the hydroxide and ligand major and minor components of the disordered ethanol solvate molecule are observed and may account for the tenacity of the ethanol binding. The HO···O distances are 2.807 (11) Å and 2.83 (2) Å and the O—H···O distances observed are 1.99 (3) and 2.03 (4) Å for the major and minor components of the disordered ethanol.

In addition to the hydrogen bonds involving the solvate molecule, N—H···O and N—H···N hydrogen bonding involving the uncoordinated amine group as the donor moiety and acetate ligands and the coordinated amine group as the acceptors are observed. Pertinent metrics involving these interactions are found in Table 1.

The benzene ring of the diamine ligand is planar with the atom having the largest deviation being C2, which sits 0.011 (6) Å above the plane. The two amine nitrogen atoms deviate only slightly from the benzene ring plane with N1 being 0.048 (13) Å and N2 being 0.005 (0.012) Å above the plane.

The identical synthetic strategy employed using symmetrically substituted diamines results in a molecular species (c.f., Geiger, 2012). We have prepared an analogue of the title compound employing 1,2-diamino-4-cyanobenzene as the diamine. The structure of the compound is virtually the same (although not isomorphous), but all attempts to obtain a structure of publishable quality have failed. Whether or not the use of unsymmetrically-substituted 1,2-diaminobenzene is a prerequisite for the formation of a Zn MOF of this structureal type is yet to be determined. We are exploring other unsymmetrically substuted diamines in hopes of better understanding this phenomenon.

### S2. Experimental

The title compound was prepared by the reaction of two equivalents of 1,2-diamino-4-chlorobenzene with zinc acetate dihydrate in refluxing ethanol. Slow evaporation of the solvent resulted in the formation of layers of extremely thin, colorless plates. The samples used for analysis were cut from carefully peeled apart layers.

### S3. Refinement

Crystal data, data collection and structure refinement details are summarized in Table 1. As a result of the unsymmetrical nature of the 1,2-diamino-4-chlorobenzene ligand, the chloro substitutent was refined with a site occupancy factor of one-half in each of two positions. The ethanol solvate is rotationally disordered over two positions. It was refined with the C—C and C—O distances restrained to 1.54 and 1.43 Å, respectively, using *DFIX*. The anisotropic displacement parameters of the minor component were constrained to the same refined values of the major component using EADP. The refined occupancies of the major and minor contributors are 0.696 (11) and 0.304 (11), respectively. The maximum shift/error for the disorder occupancies and the coordinates and anisotropic displacement parameters of the ethanol solvate were 0.000. In the final set of refinement cycles, the occupancies of the major and minor components were fixed at 0.7 and 0.3, respectively. The maximum residual electron density (0.98 e^3^) and the deepest hole (-1.05 e^3^) are located 1.10 Å and 0.86 Å, respectively, from the Zn2 atom.

All hydrogen atoms except those associated with the disordered ethanol solvent molecule were observed in difference fourier maps. The carbon-bonded hydrogen atoms were refined using a riding model with a C—H distance of 0.98 Å for the methyl carbon atoms and 0.95 Å for the phenyl carbon atoms. The atomic coordinates for the nitrogen- and oxygen-bonded hydrogen atoms were refined using the restraints *DFIX* = 0.84 Å for O—H and 0.91 Å for N—H.

The methyl C—H and the O—H hydrogen atom isotropic displacement parameters were set using the approximation *U*_iso_ = 1.5*U*_eq_. All other hydrogen atom isotropic displacement parameters were set using the approximation *U*_iso_ = 1.2*U*_eq_.

### Crystal data

**Table:**

[Zn_2_(C_2_H_3_O_2_)_3_(OH)(C_6_H_7_ClN_2_)]·C_2_H_6_O	*Z* = 2
*M**_r_* = 513.53	*F*(000) = 524
Triclinic, *P*1	*D*_x_ = 1.664 Mg m^−^^3^
*a* = 8.0769 (12) Å	Mo *K*α radiation, λ = 0.71073 Å
*b* = 10.8723 (19) Å	Cell parameters from 2325 reflections
*c* = 12.909 (3) Å	θ = 2.3–24.9°
α = 101.511 (6)°	µ = 2.51 mm^−^^1^
β = 96.399 (6)°	*T* = 200 K
γ = 109.817 (5)°	Plate, clear colourless
*V* = 1025.1 (3) Å^3^	0.60 × 0.40 × 0.02 mm

### Data collection

**Table:**

Bruker SMART X2S benchtop diffractometer	3329 independent reflections
Radiation source: XOS X-beam microfocus source	2403 reflections with *I* > 2σ(*I*)
Doubly curved silicon crystal monochromator	*R*_int_ = 0.061
Detector resolution: 8.3330 pixels mm^-1^	θ_max_ = 25.1°, θ_min_ = 2.7°
ω scans	*h* = −8→8
Absorption correction: multi-scan (*SADABS*; Bruker, 2013)	*k* = −12→12
*T*_min_ = 0.52, *T*_max_ = 0.95	*l* = −15→13
6443 measured reflections	

### Refinement

**Table:**

Refinement on *F*^2^	Primary atom site location: structure-invariant direct methods
Least-squares matrix: full	Secondary atom site location: difference Fourier map
*R*[*F*^2^ > 2σ(*F*^2^)] = 0.059	Hydrogen site location: mixed
*wR*(*F*^2^) = 0.181	H atoms treated by a mixture of independent and constrained refinement
*S* = 1.05	*w* = 1/[σ^2^(*F*_o_^2^) + (0.0797*P*)^2^ + 5.5001*P*] where *P* = (*F*_o_^2^ + 2*F*_c_^2^)/3
3329 reflections	(Δ/σ)_max_ < 0.001
280 parameters	Δρ_max_ = 0.98 e Å^−^^3^
92 restraints	Δρ_min_ = −1.05 e Å^−^^3^

### Special details

**Table:**

Geometry. All e.s.d.'s (except the e.s.d. in the dihedral angle between two l.s. planes) are estimated using the full covariance matrix. The cell e.s.d.'s are taken into account individually in the estimation of e.s.d.'s in distances, angles and torsion angles; correlations between e.s.d.'s in cell parameters are only used when they are defined by crystal symmetry. An approximate (isotropic) treatment of cell e.s.d.'s is used for estimating e.s.d.'s involving l.s. planes.
Refinement. Refinement of *F*^2^ against ALL reflections. The weighted *R*-factor *wR* and goodness of fit *S* are based on *F*^2^, conventional *R*-factors *R* are based on *F*, with *F* set to zero for negative *F*^2^. The threshold expression of *F*^2^ > σ(*F*^2^) is used only for calculating *R*-factors(gt) *etc*. and is not relevant to the choice of reflections for refinement. *R*-factors based on *F*^2^ are statistically about twice as large as those based on *F*, and *R*- factors based on ALL data will be even larger.

### Fractional atomic coordinates and isotropic or equivalent isotropic displacement parameters (Å^2^)

**Table:**

	*x*	*y*	*z*	*U*_iso_*/*U*_eq_	Occ. (<1)
Zn1	0.51291 (11)	0.64882 (9)	0.49816 (7)	0.0220 (3)	
Zn2	0.10138 (11)	0.36887 (9)	0.47758 (7)	0.0235 (3)	
O1	0.3290 (7)	0.4519 (5)	0.4368 (4)	0.0206 (12)	
H1	0.311 (11)	0.419 (8)	0.370 (2)	0.031*	
Cl1	0.1090 (8)	0.5949 (6)	0.0220 (4)	0.0601 (15)	0.5
Cl2	0.4363 (11)	0.9055 (7)	0.0139 (4)	0.087 (2)	0.5
N1	0.6409 (9)	0.9892 (7)	0.4111 (6)	0.0293 (16)	
H1A	0.744 (6)	1.047 (7)	0.399 (6)	0.035*	
H1B	0.682 (9)	0.964 (8)	0.469 (4)	0.035*	
N2	0.3623 (9)	0.7554 (7)	0.4280 (5)	0.0247 (15)	
H2A	0.242 (3)	0.719 (7)	0.422 (6)	0.03*	
H2B	0.396 (9)	0.822 (6)	0.490 (4)	0.03*	
C1	0.5200 (11)	0.8997 (8)	0.3188 (7)	0.0273 (17)	
C2	0.3789 (11)	0.7860 (8)	0.3266 (6)	0.0232 (16)	
C3	0.2564 (12)	0.6995 (9)	0.2341 (7)	0.0340 (19)	
H3	0.1629	0.6209	0.2395	0.041*	
C4	0.2699 (14)	0.7271 (11)	0.1347 (8)	0.050 (2)	
H4	0.1854	0.6683	0.0721	0.06*	0.5
C5	0.4063 (15)	0.8402 (12)	0.1269 (8)	0.051 (2)	
H5	0.4145	0.8598	0.0588	0.062*	0.5
C6	0.5331 (14)	0.9267 (10)	0.2183 (8)	0.045 (2)	
H6	0.628	1.0037	0.2117	0.054*	
O71	0.0171 (7)	0.1738 (5)	0.3941 (5)	0.0304 (14)	
O72	0.7122 (7)	0.8392 (5)	0.5729 (5)	0.0334 (14)	
C7	0.1261 (11)	0.1117 (8)	0.3829 (7)	0.030 (2)	
C71	0.0524 (14)	−0.0317 (10)	0.3175 (10)	0.062 (4)	
H71A	0.073	−0.0348	0.2438	0.093*	
H71B	−0.0767	−0.071	0.3161	0.093*	
H71C	0.1127	−0.0834	0.3498	0.093*	
O81	−0.1322 (7)	0.3856 (5)	0.4406 (4)	0.0243 (12)	
O82	0.4119 (7)	0.6770 (6)	0.6442 (4)	0.0260 (13)	
C8	−0.2514 (11)	0.3399 (7)	0.3538 (6)	0.0213 (17)	
C81	−0.1933 (12)	0.3104 (9)	0.2482 (6)	0.033 (2)	
H20A	−0.2959	0.2829	0.189	0.05*	
H20B	−0.1474	0.2373	0.2451	0.05*	
H20C	−0.0986	0.3917	0.2416	0.05*	
O91	0.1070 (7)	0.3617 (6)	0.6295 (4)	0.0265 (13)	
O92	0.3883 (8)	0.3702 (6)	0.6454 (5)	0.0360 (15)	
C9	0.2520 (12)	0.3665 (8)	0.6821 (7)	0.0277 (19)	
C91	0.2600 (14)	0.3703 (10)	0.7993 (7)	0.039 (2)	
H91A	0.355	0.3401	0.8245	0.059*	
H91B	0.1445	0.3104	0.8094	0.059*	
H91C	0.2858	0.4629	0.8406	0.059*	
OE1	0.7175 (15)	0.6924 (13)	0.7773 (8)	0.062 (3)	0.7
H1E1	0.6255	0.7008	0.7473	0.093*	0.7
C1E1	0.711 (4)	0.698 (2)	0.8858 (13)	0.103 (9)	0.7
HE1A	0.7271	0.6172	0.9035	0.124*	0.7
HE1B	0.5934	0.6975	0.9	0.124*	0.7
C2E1	0.855 (4)	0.821 (3)	0.954 (2)	0.126 (9)	0.7
HE1C	0.9712	0.8238	0.9361	0.189*	0.7
HE1D	0.8568	0.8222	1.0297	0.189*	0.7
HE1E	0.8339	0.9008	0.94	0.189*	0.7
OE2	0.719 (4)	0.591 (3)	0.7894 (18)	0.062 (3)	0.3
H1E2	0.6074	0.5492	0.7742	0.093*	0.3
C1E2	0.765 (11)	0.688 (4)	0.887 (3)	0.103 (9)	0.3
HE2A	0.8724	0.6864	0.9316	0.124*	0.3
HE2B	0.6656	0.6665	0.9267	0.124*	0.3
C2E2	0.803 (9)	0.821 (4)	0.869 (5)	0.126 (9)	0.3
HE2C	0.9333	0.8675	0.8779	0.189*	0.3
HE2D	0.7577	0.8733	0.9218	0.189*	0.3
HE2E	0.7449	0.813	0.7962	0.189*	0.3

### Atomic displacement parameters (Å^2^)

**Table:**

	*U*^11^	*U*^22^	*U*^33^	*U*^12^	*U*^13^	*U*^23^
Zn1	0.0152 (5)	0.0202 (5)	0.0324 (5)	0.0073 (4)	0.0046 (4)	0.0096 (4)
Zn2	0.0130 (5)	0.0251 (5)	0.0337 (5)	0.0071 (4)	0.0057 (4)	0.0100 (4)
O1	0.016 (3)	0.024 (3)	0.021 (3)	0.008 (2)	0.004 (2)	0.003 (2)
Cl1	0.071 (4)	0.065 (4)	0.030 (2)	0.018 (3)	−0.002 (2)	0.003 (2)
Cl2	0.130 (6)	0.073 (4)	0.033 (3)	0.008 (4)	0.005 (3)	0.020 (3)
N1	0.025 (4)	0.024 (4)	0.041 (4)	0.006 (3)	0.014 (3)	0.015 (3)
N2	0.017 (3)	0.024 (4)	0.031 (4)	0.006 (3)	0.005 (3)	0.007 (3)
C1	0.027 (4)	0.025 (4)	0.040 (4)	0.014 (3)	0.018 (3)	0.017 (3)
C2	0.023 (4)	0.021 (4)	0.035 (4)	0.015 (3)	0.012 (3)	0.012 (3)
C3	0.030 (4)	0.037 (4)	0.038 (4)	0.016 (4)	0.006 (3)	0.011 (3)
C4	0.050 (5)	0.058 (5)	0.039 (4)	0.021 (4)	0.002 (4)	0.009 (4)
C5	0.054 (5)	0.064 (5)	0.045 (4)	0.021 (4)	0.014 (4)	0.032 (4)
C6	0.047 (5)	0.046 (5)	0.048 (4)	0.013 (4)	0.019 (4)	0.026 (4)
O71	0.015 (3)	0.019 (3)	0.055 (4)	0.004 (3)	0.008 (2)	0.009 (3)
O72	0.021 (3)	0.017 (3)	0.057 (4)	0.002 (3)	−0.003 (3)	0.010 (3)
C7	0.020 (5)	0.020 (4)	0.044 (5)	0.002 (4)	0.000 (4)	0.010 (4)
C71	0.033 (6)	0.028 (6)	0.101 (9)	0.010 (5)	−0.014 (6)	−0.019 (6)
O81	0.017 (3)	0.026 (3)	0.026 (3)	0.008 (2)	0.000 (2)	0.003 (2)
O82	0.019 (3)	0.030 (3)	0.028 (3)	0.010 (3)	0.001 (2)	0.004 (2)
C8	0.025 (4)	0.016 (3)	0.029 (3)	0.012 (3)	0.006 (3)	0.011 (3)
C81	0.029 (5)	0.043 (5)	0.032 (4)	0.019 (4)	0.010 (4)	0.010 (4)
O91	0.022 (3)	0.030 (3)	0.033 (3)	0.012 (3)	0.007 (2)	0.014 (2)
O92	0.034 (4)	0.048 (4)	0.044 (3)	0.025 (3)	0.023 (3)	0.023 (3)
C9	0.032 (5)	0.018 (4)	0.040 (5)	0.015 (4)	0.008 (4)	0.010 (4)
C91	0.050 (6)	0.041 (5)	0.034 (5)	0.023 (5)	0.012 (4)	0.012 (4)
OE1	0.057 (6)	0.092 (8)	0.044 (5)	0.044 (6)	−0.001 (4)	0.009 (5)
C1E1	0.103 (11)	0.106 (10)	0.102 (9)	0.048 (6)	0.009 (6)	0.021 (6)
C2E1	0.135 (14)	0.120 (13)	0.110 (13)	0.030 (11)	0.007 (11)	0.041 (11)
OE2	0.057 (6)	0.092 (8)	0.044 (5)	0.044 (6)	−0.001 (4)	0.009 (5)
C1E2	0.103 (11)	0.106 (10)	0.102 (9)	0.048 (6)	0.009 (6)	0.021 (6)
C2E2	0.135 (14)	0.120 (13)	0.110 (13)	0.030 (11)	0.007 (11)	0.041 (11)

### Geometric parameters (Å, º)

**Table:**

Zn1—O1	2.079 (5)	C7—C71	1.494 (12)
Zn1—O92^i^	2.098 (6)	C71—H71A	0.98
Zn1—O72	2.098 (5)	C71—H71B	0.98
Zn1—O82	2.143 (5)	C71—H71C	0.98
Zn1—O1^i^	2.147 (5)	O81—C8	1.281 (9)
Zn1—N2	2.185 (7)	O82—C8^iv^	1.251 (9)
Zn2—O1	1.937 (5)	C8—O82^iv^	1.251 (9)
Zn2—O81	1.971 (5)	C8—C81	1.500 (11)
Zn2—O91	1.974 (5)	C81—H20A	0.98
Zn2—O71	2.017 (5)	C81—H20B	0.98
O1—Zn1^i^	2.147 (5)	C81—H20C	0.98
O1—H1	0.84 (2)	O91—C9	1.266 (10)
Cl1—C4	1.830 (11)	O92—C9	1.237 (10)
Cl1—Cl1^ii^	2.128 (12)	O92—Zn1^i^	2.098 (6)
Cl2—C5	1.752 (11)	C9—C91	1.499 (12)
Cl2—Cl2^iii^	2.086 (14)	C91—H91A	0.98
N1—C1	1.398 (11)	C91—H91B	0.98
N1—H1A	0.91 (2)	C91—H91C	0.98
N1—H1B	0.91 (2)	OE1—C1E1	1.398 (15)
N2—C2	1.423 (10)	OE1—H1E1	0.84
N2—H2A	0.90 (2)	C1E1—C2E1	1.474 (17)
N2—H2B	0.90 (2)	C1E1—HE1A	0.99
C1—C6	1.394 (12)	C1E1—HE1B	0.99
C1—C2	1.400 (11)	C2E1—HE1C	0.98
C2—C3	1.395 (11)	C2E1—HE1D	0.98
C3—C4	1.383 (13)	C2E1—HE1E	0.98
C3—H3	0.95	OE2—C1E2	1.39 (2)
C4—C5	1.375 (15)	OE2—H1E2	0.84
C4—H4	0.95	C1E2—C2E2	1.45 (3)
C5—C6	1.399 (14)	C1E2—HE2A	0.99
C5—H5	0.95	C1E2—HE2B	0.99
C6—H6	0.95	C2E2—HE2C	0.98
O71—C7	1.282 (10)	C2E2—HE2D	0.98
O72—C7^i^	1.248 (10)	C2E2—HE2E	0.98
C7—O72^i^	1.248 (10)		
			
O1—Zn1—O92^i^	88.2 (2)	C7—O71—Zn2	121.6 (5)
O1—Zn1—O72	173.5 (2)	C7^i^—O72—Zn1	138.8 (6)
O92^i^—Zn1—O72	94.2 (3)	O72^i^—C7—O71	124.4 (8)
O1—Zn1—O82	93.6 (2)	O72^i^—C7—C71	117.7 (8)
O92^i^—Zn1—O82	177.4 (2)	O71—C7—C71	117.8 (7)
O72—Zn1—O82	84.2 (2)	C7—C71—H71A	109.5
O1—Zn1—O1^i^	82.3 (2)	C7—C71—H71B	109.5
O92^i^—Zn1—O1^i^	91.3 (2)	H71A—C71—H71B	109.5
O72—Zn1—O1^i^	91.6 (2)	C7—C71—H71C	109.5
O82—Zn1—O1^i^	90.8 (2)	H71A—C71—H71C	109.5
O1—Zn1—N2	99.1 (2)	H71B—C71—H71C	109.5
O92^i^—Zn1—N2	86.4 (2)	C8—O81—Zn2	132.6 (5)
O72—Zn1—N2	87.1 (2)	C8^iv^—O82—Zn1	123.4 (5)
O82—Zn1—N2	91.5 (2)	O82^iv^—C8—O81	121.4 (7)
O1^i^—Zn1—N2	177.3 (2)	O82^iv^—C8—C81	120.2 (7)
O1—Zn2—O81	130.8 (2)	O81—C8—C81	118.4 (7)
O1—Zn2—O91	117.1 (2)	C8—C81—H20A	109.5
O81—Zn2—O91	101.1 (2)	C8—C81—H20B	109.5
O1—Zn2—O71	103.6 (2)	H20A—C81—H20B	109.5
O81—Zn2—O71	94.7 (2)	C8—C81—H20C	109.5
O91—Zn2—O71	104.5 (2)	H20A—C81—H20C	109.5
Zn2—O1—Zn1	127.8 (2)	H20B—C81—H20C	109.5
Zn2—O1—Zn1^i^	101.7 (2)	C9—O91—Zn2	117.2 (5)
Zn1—O1—Zn1^i^	97.7 (2)	C9—O92—Zn1^i^	143.2 (6)
Zn2—O1—H1	105 (6)	O92—C9—O91	125.6 (8)
Zn1—O1—H1	117 (6)	O92—C9—C91	117.3 (8)
Zn1^i^—O1—H1	103 (6)	O91—C9—C91	117.1 (8)
C4—Cl1—Cl1^ii^	144.8 (6)	C9—C91—H91A	109.5
C5—Cl2—Cl2^iii^	136.3 (6)	C9—C91—H91B	109.5
C1—N1—H1A	115 (5)	H91A—C91—H91B	109.5
C1—N1—H1B	125 (5)	C9—C91—H91C	109.5
H1A—N1—H1B	102 (4)	H91A—C91—H91C	109.5
C2—N2—Zn1	122.9 (5)	H91B—C91—H91C	109.5
C2—N2—H2A	102 (5)	C1E1—OE1—H1E1	109.5
Zn1—N2—H2A	115 (6)	OE1—C1E1—C2E1	109.4 (18)
C2—N2—H2B	121 (6)	OE1—C1E1—HE1A	109.8
Zn1—N2—H2B	92 (5)	C2E1—C1E1—HE1A	109.8
H2A—N2—H2B	102 (4)	OE1—C1E1—HE1B	109.8
C6—C1—N1	120.2 (8)	C2E1—C1E1—HE1B	109.8
C6—C1—C2	119.1 (8)	HE1A—C1E1—HE1B	108.2
N1—C1—C2	120.6 (7)	C1E1—C2E1—HE1C	109.5
C3—C2—C1	120.0 (8)	C1E1—C2E1—HE1D	109.5
C3—C2—N2	119.5 (7)	HE1C—C2E1—HE1D	109.5
C1—C2—N2	120.5 (7)	C1E1—C2E1—HE1E	109.5
C4—C3—C2	120.6 (9)	HE1C—C2E1—HE1E	109.5
C4—C3—H3	119.7	HE1D—C2E1—HE1E	109.5
C2—C3—H3	119.7	C1E2—OE2—H1E2	109.5
C5—C4—C3	119.7 (9)	OE2—C1E2—C2E2	111 (3)
C5—C4—Cl1	126.1 (8)	OE2—C1E2—HE2A	109.5
C3—C4—Cl1	114.0 (8)	C2E2—C1E2—HE2A	109.5
C5—C4—H4	120.2	OE2—C1E2—HE2B	109.5
C3—C4—H4	120.2	C2E2—C1E2—HE2B	109.5
C4—C5—C6	120.8 (9)	HE2A—C1E2—HE2B	108.1
C4—C5—Cl2	127.8 (9)	C1E2—C2E2—HE2C	109.5
C6—C5—Cl2	111.2 (8)	C1E2—C2E2—HE2D	109.5
C4—C5—H5	119.6	HE2C—C2E2—HE2D	109.5
C6—C5—H5	119.6	C1E2—C2E2—HE2E	109.5
C1—C6—C5	119.9 (9)	HE2C—C2E2—HE2E	109.5
C1—C6—H6	120.1	HE2D—C2E2—HE2E	109.5
C5—C6—H6	120.1		

Symmetry codes: (i) −*x*+1, −*y*+1, −*z*+1; (ii) −*x*, −*y*+1, −*z*; (iii) −*x*+1, −*y*+2, −*z*; (iv) −*x*, −*y*+1, −*z*+1.

### Hydrogen-bond geometry (Å, º)

**Table:**

*D*—H···*A*	*D*—H	H···*A*	*D*···*A*	*D*—H···*A*
O1—H1···O*E*1^i^	0.84 (2)	1.99 (3)	2.807 (11)	166 (8)
O1—H1···O*E*2^i^	0.84 (2)	2.03 (4)	2.83 (2)	160 (8)
N2—H2*A*···O81^iv^	0.90 (2)	2.38 (7)	2.914 (9)	118 (6)
N2—H2*B*···N1^v^	0.91 (6)	2.32 (5)	3.128 (10)	148 (7)
N1—H1*A*···O71^vi^	0.91 (7)	2.19 (3)	3.082 (9)	169 (6)
N1—H1*B*···O72	0.91 (6)	2.14 (7)	3.013 (9)	160 (7)
O*E*1—H1*E*1···O82	0.84	1.97	2.780 (11)	163
O*E*2—H1*E*2···O92	0.84	2.32	3.02 (3)	140

Symmetry codes: (i) −*x*+1, −*y*+1, −*z*+1; (iv) −*x*, −*y*+1, −*z*+1; (v) −*x*+1, −*y*+2, −*z*+1; (vi) *x*+1, *y*+1, *z*.
